# Research progress of endogenous neural stem cells in spinal cord injury

**DOI:** 10.1002/ibra.12048

**Published:** 2022-06-04

**Authors:** Ya‐Ting Wang, Hao Yuan

**Affiliations:** ^1^ Department of Anesthesiology Southwest Medical University Luzhou Sichuan China; ^2^ Institute of Neuroscience Kunming Medical University Kunming Yunnan China

**Keywords:** endogenous neural stem cells, influencing factor, spinal cord injury, therapy

## Abstract

Spinal cord injury (SCI) is a severe disabling disease, which mainly manifests as impairments of sensory and motor functions, sexual function, bladder and intestinal functions, respiratory and cardiac functions below the injury plane. In addition, the condition has a profound effect on the mental health of patients, which often results in severe sequelae. Some patients may be paraplegic for life or even die, which places a huge burden on the family and society. There is still no effective treatment for SCI. Studies have confirmed that endogenous neural stem cells (ENSCs), as multipotent neural stem cells, which are located in the ependymal region of the central canal of the adult mammalian spinal cord, are activated after SCI and then differentiate into various nerve cells to promote endogenous repair and regeneration. However, the central canal of the spinal cord is often occluded to varying degrees in adults, and residual ependymal cells cannot be activated and do not proliferate after SCI. Besides, the destruction of the microenvironment after SCI is also an important factor that affects the proliferation and differentiation of ENSCs and spinal cord repair. Therefore, this review describes the role of ENSCs in SCI, in terms of the origin, transformation, treatment, and influencing factors, to provide new ideas for clinical treatment of SCI.

## Graphical abstract

Endogenous neural stem cells (ENSCs) in the ependymal region of the spinal cord of adult mammals may play a key role in endogenous repair and regeneration. But there are no ENSCs in the adult spinal cord, this may way many therapeutic approaches succeed on the experiments but fail in the clinic. How to activate ENSCs and guide them to migrate to the injury site to proliferate and differentiate into neurons with repair function is the current research hot topic, but how to determine the optimal treatment plan from the numerous influencing factors is a problem. This article reviews the research progress of ENSCs in spinal cord injury from the aspects of origin, transformation, treatment, and influencing factors.

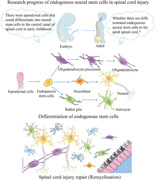

## 1 INTRODUCTION

Spinal cord injury (SCI) is the most severe complication of spine injury; it can be divided into traumatic SCI and nontraumatic SCI according to the etiology.^1^ Traumatic SCI involves vertebral body or bone dislocation, caused by external physical impact.^2^ Nontraumatic SCI includes developmental SCI and acquired SCI. Developmental SCI is a congenital malformation of spinal cord blood vessels that causes blood vessels to rupture and hemorrhage, and compresses the spinal cord to cause injury. Acquired SCI is caused by spinal cord tumor, myelitis, and other lesions that compress the spinal cord tissue, leading to spinal cord ischemia and hypoxia, resulting in SCI.^3^

The global morbidity of SCI was between 10.4 and 83 million cases per year, and the age‐standardized incidence was 13 (11–16)/100,000. In 2016 alone, SCI resulted in 9.5 (6.7–12.4) million disability lives.^4^ The onset age of SCI showed a bimodal distribution, with young males aged 15–29 years in the first peak and the elderly in the second peak. The average age of onset was 40 years. A large proportion of young and middle‐aged people lose their ability to work, which is not conducive to the development of society.^5^ It has been estimated that the acute hospitalization mortality rate is between 4% and 17%. Even after discharge, the annual death rate remains high. Besides, at 1 year after injury, only 12% of persons with SCI are employed, and by 20 years postinjury, about one‐third is employed, which places a huge burden on families and society.^6^

There are many treatment methods for SCI, among which the management strategies for acute SCI include early surgical decompression and fixation. However, the therapeutic effect of SCI is still unsatisfactory.^7^, ^8^ Stem cells have received considerable attention from the medical community due to their potential to regenerate various tissues and organs. Although there are many challenges in the transition from the laboratory to the clinic in terms of treatment of stem cell transplantation, such as the potential tumorigenic risk of undifferentiated human induced pluripotent stem cells, and the technical requirements for large‐scale culture after clinical treatment, and so forth,^9^, ^10^ stem cells still have great potential in the treatment of neurological diseases. Endogenous neural stem cells (ENSCs) are a group of cells with the potential to differentiate; these are located in the ependymal membrane of the spinal cord. They are functionally inactive under normal conditions, and activated as functional stem cells under the stimulation of SCI, which may be a potential therapeutic strategy for spinal cord recovery.

ENSCs are multifunctional cells that can self‐renew, which can divide and differentiate into various types of nerve cells through both symmetric and asymmetric cell division. Differentiated nerve cells can migrate to sites requiring repair to replace damaged neurons, enabling treatment of serious neurological diseases such as neurodegeneration, stroke, and SCI.^11^, ^12^ SCI leads to axonal separation, neuronal death, and permanent functional impairment. Stem cell therapy can not only replenish lost neurons and connect disrupted axonal pathways but also secrete neurotrophic factors to promote axonal regeneration. Stem cells, such as ENSCs, are a hot research topic in the treatment of SCI.^13^ In addition, as a new type of treatment, ENSCs can avoid immune rejection and ethical issues, which have broad research and application prospects.^14^ Therefore, this article reviews the research progress of ENSCs in SCI from the aspects of origin, transformation, treatment, and influencing factors, to provide new ideas for clinical treatment of SCI.

## 2 ORIGIN AND DISTRIBUTION OF ENSCs

The ependymal membrane of the lateral recess of the fourth ventricle and the central canal of the spinal cord in mammals is a relatively static pool of adult ENSCs. The neurogenic activities of ENSCs run through the whole life cycle and show stem cell characteristics after SCI.^15^

**Figure 1:**
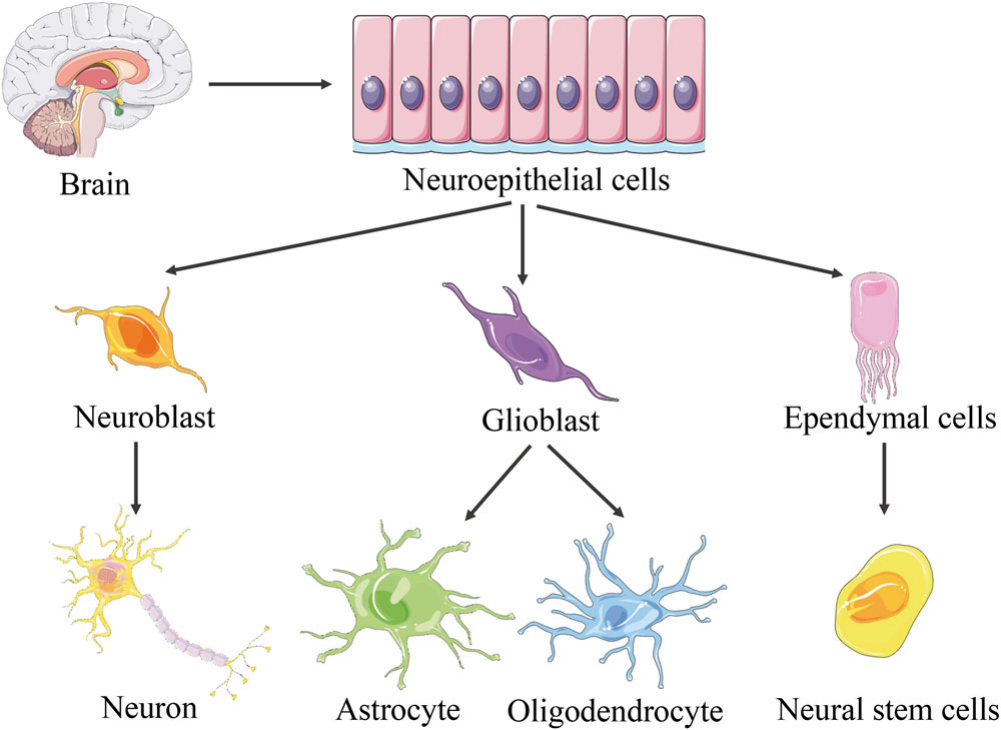
Origin process diagram of endogenous neural stem cells. [Color figure can be viewed at wileyonlinelibrary.com]

Spinal ependymal cells are derived from neuroepithelial cells. Neuroepithelial cells in the cerebral ventricle region differentiate into neuroblasts, and give rise to neurons. Neuroepithelial cells can also differentiate into glioblasts, astrocytes, and oligodendrocytes. The remaining neuroepithelial cells differentiate into ependymal cells that line the myelocoele.^16^, ^17^ Ependyma is composed of three main cell types: elongation cell, ependymal cells, and cerebrospinal fluid contact neurons.^18^ The ependymal cells remain relatively static in the healthy state, and show neural stem cell characteristics in various disease states (Figure 1).

## 3 TRANSFORMATION OF EPENDYMAL CELLS

The ependymal cells of rats went through two developmental stages and finally distributed in the ventricular region of the spinal cord. Most ependymal cells emerged from about 18 days of the embryo stage, and a few began to develop between 8 and 15 days after birth. In the ventricular region of the spinal cord, ependymal cell proliferation and production continue at a slower rate until at least 36 days after birth.^19^

For humans, however, the central canals usually gradually disappear from early childhood, replaced by astrocyte proliferation, ependymal clusters, and perivascular pseudoknots. Yasui et al.^20^ studied the age‐related morphologic changes in the normal human central canal of the spinal cord from 158 autopsy cases ranging in age from 1 week postnatally to 116 years of age. The results showed that the patency rate of each segment of the spinal cord central canal was almost 100% under 1 year of age, with significant occlusion occurring after 20 years of age and increasing with age. However, this finding has been questioned and ignored because the study was conducted on cadavers. Also, by using magnetic resonance imaging, laser capture microscopy, histology, and immunohistochemistry to evaluate the central canal in control subjects and SCI patients, Garcia‐Ovejero et al.^21^ reported that the unobstructed central canal is absent from the vast majority of individuals beyond the age of 18 years throughout the entire length of the spinal cord after injury.

In human ependymal residues, ependymal cells do not proliferate and differentiate under normal conditions. After studying the human ependymal remnant from 21 individuals with traumatic SCI survival times ranging from days to months postinjury, Paniagua‐Torija et al.^22^ demonstrated that the ependymal residues of adults will not proliferate at any time or distance from the lesion after injury.

In conclusion, ependymal cells have always existed in the spinal cord of adult mammals such as rats, but the central canal of the spinal cord is occluded to varying degrees in adults, and studies have proved that ependymal residue does not proliferate after SCI. This may be the reason why the treatment of SCI with ENSCs has made smooth progress in animal research, but has not been successful in clinical translation.

## 4 ENSC THERAPY ON SCI

SCI leads to cell damage, axon separation, and neuron death. Damaged nerve cells, axons, and vascular endothelial cells release harmful substances that attack adjacent tissues, leading to permanent dysfunction.^23^ In addition, the SCI blocks supraspinal information to reach the peripheral muscles, leading to motor function loss, and also blocks peripheral information from reaching the brain, leading to sensory function loss.^24^

After developing a model with minimal SCI and labeling the ependymal by injecting markers, some studies found that the proliferation marker index of ependymal cells increased significantly and most of the cells migrated within 70 microns of the central canal. These results showed that minimal SCI near the ependymal was sufficient to induce ependymal cell proliferation and migration, which was considered a promising cell source for therapeutic nerve repair.^25^ After activation by SCI, ependymal cells begin to divide and differentiate into ENSCs, which in turn differentiate into astrocytes, oligodendrocytes, and neurons.^26^ New neurons can replace damaged neurons, repair the functional damage caused by the death of neurons, and form synapses to rebuild neural circuits. Oligodendrocytes can promote the myelination of new nerve fibers and the remaining demyelinating fibers, to restore the integrity of nerve fibers.^27^ In addition, ENSCs can produce a variety of extracellular matrices that fill the gaps left after injury and provide support for neuronal axonal regeneration.

ENSCs differentiated into a large number of new neurons, including various intermediate motor neurons and sensory neurons, which send axons into normal spinal cord tissue to establish axonal connections with downstream neurons. At the same time, ENSCs can promote the regeneration of descending motor axons and ascending sensory axons in injured spinal cord. The regenerated axons established functional axonal connections with newborn neurons, and thus reconstructed the continuity of the spinal nerve signaling pathway.^28^

**Figure 2:**
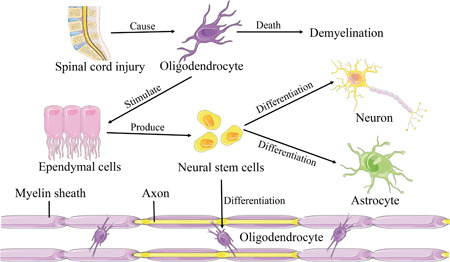
Demyelination and remyelination after spinal cord injury. [Color figure can be viewed at wileyonlinelibrary.com]

Myelin regeneration plays an important role in axon function recovery after SCI. SCI is often accompanied by a long period of slow death of oligodendrocytes, which is an important cause of widespread demyelination.^29^, ^30^ Existing evidence suggests that in animal models with extensive demyelination of the central nervous system (CNS) but oligodendrocyte survival, oligodendrocytes were linked to mature and regenerated myelin sheaths during convalescence. These results indicated that oligodendrocytes maintained mature myelin, re‐extended the axonal connections between demyelinating myelin, and participated in myelin regeneration.^31^, ^32^ After activation, ependymal cells differentiate into ENSCs, and then generate oligodendrocytes. This, to some extent, compensates for the death of oligodendrocytes caused by SCI, and promotes myelin regeneration and spinal cord recovery (Figure 2).

The extracellular matrix of the spinal cord contains structural and communication proteins involved in repair and regeneration after SCI.^33^, ^34^ They not only provide support for neuronal regeneration but also achieve homeostasis through receptor or receptor‐mediated action and the related positioning of other signal molecules in time and space, and have a positive regulatory role and signal conduction function.^35^ Liu et al.^36^ linked the lumen after SCI by a “cocktail” of a functional self‐assembling peptide (F‐SAP) nanofiber hydrogel with growth factors, and it was found that this promoted the formation of connections to the severed corticospinal tract and myelination by recombining the extracellular matrix.^36^ These results indicated that the extracellular matrix plays an important role in the functional recovery after SCI. Activated ENSCs generate various extracellular matrices, which provide support for neuronal axonal regeneration and promote signal connection between nerve cells and the functional recovery of SCI.

## 5 INFLUENCING FACTORS OF ENSC THERAPY FOR SCI

### 5.1 Influencing factors of activation of ENSCs

Nestin is a member of the intermediate fiber family, and its expression increases in a variety of tumor tissues. Its protein expression level is positively correlated with the degree of malignancy of tumor, so the expression level of Nestin in tumor tissues is of great significance for evaluating the biological behavior of tumors and the prognosis of patients.^37^ In addition, Nestin is highly expressed in mammalian neural progenitor cells and is widely used as a marker molecule of neural progenitor cells.^38^ In transgenic mice, independent cell‐type‐specific elements in the first and second introns of the Nestin gene consistently directed reporter gene expression to developing muscle and nerve precursors, respectively. The second intron contains enhancers that function in CNS stem cells, suggesting the possible existence of a single transcriptional mechanism that regulates CNS stem cell status.^39^ Shu et al.^40^ used Nestin as a marker to isolate potential ENSCs and their direct descendants, and characterized cells before and after SCI by single‐cell RNA sequencing, and found that subpopulations located outside the central canal were activated and showed active NSC attributes after SCI. Studies have shown that Nestin(+) cells outside the central canal are activated NSCs on SCI and may be used as ENSCs in SCI regeneration therapy in the future.

The *Sox* gene family is a gene family composed of SRY‐related genes, which is highly conserved in evolution. *Sox* genes not only participate in early embryo development and neural development but also play an important role in sex determination and development during ontogeny. Previous studies have shown that *Sox2* is expressed in multipotent neural stem cells at all stages of mouse ontogeny, which may meet the requirements of ENSC activation markers.^41^ Ma et al.,^42^ by analyzing the expression of *Sox2* under the conditions of extensive passage, hypoxia culture, and differentiation, confirmed that the presence of these marker proteins is directly related to the ability of ENSCs to form new neural spheres and differentiate into multiple cell types. In addition, *Sox11* has been shown to induce the activation of ENSCs to form neurons in the injured spinal cord. Guo et al.^43^ introduced a lentivirus vector containing the *Sox11* gene into a mouse SCI model and found that *Sox11* significantly improved motor function recovery after SCI, accompanied by upregulation of Nestin expression. *Sox11* is mainly located in ENSCs of the central canal and newborn neurons of the spinal cord. Similar results can also be observed in adult zebrafish SCI models.^44^ These results indicate that *Sox11* induces the activation of ENSCs and promotes neural regeneration.

### 5.2 Influencing factors of proliferation of ENSCs

Many factors affect the proliferation of ENSCs in mice, including neurotrophic factors, hormones, physical stimulation, and so forth.

#### 5.2.1 Neurotrophic factors

Neurotrophic factors are proteins produced by nerve‐innervated tissues and astrocytes, which are necessary for the growth and development of nerve cells. Oudega et al.^45^ inserted a tube loaded with chitosan carrier neurotrophic factor‐3 (NT‐3) into a 5 mm transverse space in the spinal cord of adult rats and found that the neural tissues of Nestin (+), Tuj1 (+), and Neun (+) cells bridged the transverse space. It was shown that NT‐3 implants can induce endogenous nerve cell activation and proliferation.^45^ Functional gelatin sponge scaffolds with good porosity were extensively released in vitro after modification with NT‐3/silk fibroin complexes. After the placement of a stent in a canine SCI model, the function of the hind limb was significantly improved, and new neurons with conduction function and functional blood vessels were found in the regenerated tissue.^46^ The receptor for the ciliary neurotrophic factor (CNTF) is expressed in the subventricular region of adults. Emsley et al.^47^ injected CNTF into the forebrain of adult mice, and found that the proportion of NeuN (+)/BrdU (+) cells in the dentate gyrus and subependymal regions of the brain was increased in both mice, but did not affect differentiation into astrocytes or oligodendrocytes. The results suggest that endogenous CNTF regulates adult neurogenesis by increasing the proliferation of neural stem cells and/or precursors. Besides, the sodium hyaluronate—CNTF scaffold can activate ENSCs, and help them form glutamate‐excitatory functional synapses.^48^

#### 5.2.2 Hormones

Melatonin is a neuroendocrine hormone secreted mainly by the pineal gland. It has a variety of biological functions and neuroprotective effects, including the control of sleep–wake cycles, seasonal reproduction and body temperature, and prevention of neuronal cell death induced by neurotoxic substances. After treating SCI rats with melatonin, the proliferation and differentiation of ENSCs were detected by flow cytometry, and the level of regenerating neurons increased significantly. These findings indicated that melatonin could effectively improve the recovery of injured function by improving the proliferation and differentiation of ENSCs after SCI.^49^ Studies have found that melatonin can promote the proliferation of neural stem cells by reducing the inhibitory effect of interleukin‐18 on the proliferation of neural stem cells, the formation of neural spheres and their differentiation into neurons, and enhance the production of midbrain‐derived and glial cell‐derived neurotrophic factor.^50^ Chen et al.^51^ studied the effects of different melatonin treatment regimens on the proliferation of ENSCs. The results show that single‐dose instant melatonin treatment and continuous 7 days of melatonin treatment can promote the proliferation of ENSCs, but 7 days of continuous melatonin treatment than single‐dose instant melatonin treatment yield better effects. In addition, melatonin combined with exercise training had a synergistic effect on functional recovery after SCI. A study established a rat model of SCI, using melatonin twice a day and exercising on a treadmill for 15 min, 6 days a week for 3 weeks. Histological examination and motor function examination revealed significant improvement in hind limb function, decreased extent of myelopathy, and increased density of dendritic spines and axons at 14 and 21 days after injury. The results showed that melatonin combined with exercise training can promote the recovery of tissue and behavioral function after SCI by promoting the proliferation of ENSCs.^52^

Methylprednisolone is a synthetic steroid hormone with strong immunosuppressive effects, which can restrain the activation of ENSCs after SCI. Ye et al.^53^ injected methylprednisolone (30 mg/kg) intravenously into cynomolgus monkeys immediately after SCI, and the result showed that the percentages of *BrdU* (+) ependymal cells and Nestin (+) cells in the methylprednisolone treatment group were obviously lower than those in the SCI group. When spinal cord‐derived ENSCs were cultured in vitro under hypoxia and treated with methylprednisolone, hypoxia‐inducible factor‐1 α (HIF‐1α), and Hes1, the downstream protein of the Notch signaling pathway was inhibited. These results suggest that methylprednisolone may inhibit the proliferation and differentiation of spinal cord‐derived neural stem cells cultured under hypoxia by inhibiting HIF‐1α and Hes1.^54^

#### 5.2.3 Physical stimulus

The pulse electromagnetic field generated by electroacupuncture can effectively polarize neurons, increase enzyme activity, enhance axon transport, and facilitate regeneration of damaged neurons. Zhang et al.^55^ established a rat model of focal cerebral ischemia and reperfusion and treated with electroacupuncture, and found that electroacupuncture promoted NeuroD1‐mediated differentiation of ENSCs through exosome miR‐146b to improve nerve injury after ischemic stroke. Wu et al.^56^ treated a rat model of spinal cord contusion at T8‐9 with electroacupuncture stimulation and labeled ENSCs with BrdU and NG2. Double immunofluorescence staining showed that the number of BrdU(+)/NG2(+) cells was significantly increased in the spinal cord tissue 15 mm from the injury center. These results suggest that electroacupuncture can promote the proliferation of ENSCs and oligodendrocytes in rats with SCI. Moreover, extracorporeal shock waves (ESWs) can also promote the proliferation of ENSCs. Rats were treated with ESWs at 4 weeks after SCI, and the results showed that ENSCs proliferated significantly at the 6th week after injury and mainly distributed in the ependymal layer of the central duct and the injured posterior horn.^57^

### 5.3 Effects of microenvironment construction on ENSC therapy for SCI

The destruction of the microenvironment after SCI resulted in the differentiation of most active ENSCs into astrocytes, forming glial scar, and just the few remaining cells differentiated into neurons. The reconstruction of the microenvironment plays a crucial role in the repair of SCI.

#### 5.3.1 Collagen scaffolds

After SCI, axons and myelin sheaths break down, and even the cell body of neurons dies, resulting in progressive loss of nerve tissue and the formation of cavities or scars at the site of injury. It is necessary to implant the ideal biological scaffold into the lesion space as physical support and improve the microenvironment to promote nerve regeneration. Linear ordered collagen scaffolds (LOCSs) promote nerve regeneration, prevent scarring, and carry drugs to neutralize inhibitory molecules. In addition, different substances can be used to modify collagen scaffolds to achieve different therapeutic effects.

Studies have shown that the implantation of a LOCS modified with cetuximab at the site of SCI in dogs can lead to neuronal physiological regeneration, myelin regeneration, and functional synapse formation.^58^ N‐cadherin‐modified LOCS promoted the adhesion of ENSCs to collagen scaffolds, recruitment to injury centers, and differentiation into neurons.^59^ Myelin‐related inhibitors (MAIs) enhanced the expression of epidermal growth factor receptor (EGFR) signaling, thereby inhibiting neuronal regeneration. LOCSs modified with anti‐EGFR antibodies weaken the inhibitory effect of MAIs, induce endogenous and transplanted neural stem cells to generate new neurons, rebuild neuronal relay to promote electrical activity signal transmission, and ultimately improve damage repair.^60^ CBD‐Fab was a collagen‐bound EGFR antibody Fab fragment that neutralizes myelin suppressor molecules. Transplantation of CBD‐Fab‐modified LOCSs into a rat model of SCI revealed that ENSCs activated by endogenous injury had strong neurogenesis ability and developed into functional mature neurons to re‐establish neural connections.^61^

Through the *P38 MAPK* signaling pathway, paclitaxel (PTX)‐promoted ENSCs differentiate into neurons when the microenvironment‐promoting myelin regeneration was destroyed, and had the potential to stabilize microtubules, enhance axon regeneration, and reduce scar formation after SCI.^62^ By using a novel dual biospecificity peptide, Zhan et al.^63^ designed a PTX‐delivered MExos–collagen scaffold. MExos causes ENSCs to accumulate in injured tissues and PTX accelerates ENSCs to differentiate into neurons to promote nerve regeneration and reduce scar deposition. However, axon recovery, nerve regeneration, electrical conduction, and functional rehabilitation were promoted by the implantation of LOCS joint Taxol scaffolds only in the first scar tissue removal operation, but not in the second operation.^62^ Cetuximab is an *EGFR* signal block inhibitor. It can promote NSC to neuronal differentiation. Cetuximab‐ and PTX‐modified LOCS significantly promoted neural regeneration and reconnection of neural networks. Combined treatment with collagen scaffold provided a potential therapeutic strategy for improving functional restoration of SCI.^64^

#### 5.3.2 Hydrogel scaffolds

DNA hydrogel has extremely high permeability, can repair 2 mm of spinal cord space in rats, and create new neural networks by stimulating the regeneration of nerve cells.^65^ The CS‐HEC‐Col/GP hydrogel was a novel thermosensitive composite hydrogel based on chitosan, hydroxyethyl cellulose, collagen, and β‐glyceryl phosphate. By secreting neurotrophic factors and inhibiting apoptosis, the BMSC‐loaded CS‐HEC‐Col/GP hydrogel improved the survival or proliferation of ENSCs.^66^ The conductive polymer hydrogel based on plant‐derived polyphenols, tannic acid, cross‐linked, and doped conductive polypyrrole chain has high electrical conductivity and can promote the physiological regeneration of neurons and reduce the differentiation of ENSCs into astrocytes. In mice with severe SCI, ENSC neurogenesis in the lesion area was activated and motor function was significantly recovered.^67^ The acellular tissue matrix significantly promoted neural tissue regeneration, especially in the neural system. Compared with the decellularized matrix hydrogel extracted from the peripheral nerves, the acellular tissue matrix hydrogel extracted from the spinal cord enhanced the survival, proliferation, and migration of ENSCs by regulating the expression profile of integrins α2, α9, and β1, the AKT/ERK‐related signaling pathway, and extracellular matrix proteins, and then promoted the differentiation of neural stem/progenitor cells into neurons.^68^

#### 5.3.3 Other scaffolds

Multichannel nerve ducts were of vital importance in axon regeneration and functional recovery, and their micro/nanostructures were effective in regulating injury‐induced responses. Some researchers implanted ladder‐like porous channel walls (LNCs) and nanofibrous channel walls (NNCs) in rats with total spinal cord transection. The in vivo features showed significantly reduced inflammation and scarring, and increased recruitment of ENSCs and nerve fiber growth after injury.^69^

From what has been discussed above, bioactive scaffolds can establish an effective regeneration microenvironment for nerve cells (Table 1) and provide a potential new idea for the clinical treatment of SCI.

**Table 1:** Literature on microenvironment construction on ENSCs

Author	Years	Description	GLOST	Result
Li et al.	2017	LOCS	Cetuximab	Neuronal regeneration at the site of SCI in dogs includes neuronal differentiation, maturation, myelination, and synapse formation.
Liu et al.	2020	LOCS	N‐cadherin	LOCS‐Ncad can recruit endogenous NSPC to injury centers and promote neuronal differentiation.
Zhao et al.	2017	Collagen scaffold	Anti‐*EGFR* antibody	Scaffold‐linked antibodies induce endogenous and transplanted ENSCs to generate new neurons, re‐establish neuronal connections, and transmit electrophysiological signals.
Fan et al.	2017	LOCS	CBD‐Fab	ENSCs are strongly activated by injury.
Yin et al.	2021	LOCS	Taxol	The LOCS joint Taxol scaffold implant promotes paclitaxon recovery, nerve regeneration, electrical conduction, and functional rehabilitation in the first scar tissue removal operation.
Zhang et al.	2021	MExos–collagen scaffold	PTX	MExos recruited endogenous ENSCs to the injured site, and PTX‐induced ENSCs to produce neurons.
Fan et al.	2018	LOCS	Cetuximab + Taxol	Composite functional stent implantation can significantly promote nerve regeneration and reduce the deposition of various scar‐related inhibitors in the center of the disease.
Yuan et al.	2021	DNA hydrogel	Nothing	The DNA hydrogel was highly permeable and repaired 2 mm of spinal space in Sprague–Dawley rats.
Zhang et al.	2020	CS‐HEC‐Col/GP hydrogel	Nothing	The CS‐HEC‐COL/GP hydrogel loaded with BMSCS can secrete neurotrophic factors to inhibit cell apoptosis, thereby increasing the survival rate of nerve cells.
Zhou et al.	2018	CPH	Nothing	CPH with high electrical conductivity can activate ENSC nerves and transform them into functional neurons, reducing astrocyte transformation.
Xu et al.	2021	The acellular tissue matrix hydrogel extracted from the spinal cord	Nothing	By regulating the expression profile of integrins α2, α9, and β1, the AKT/ERK‐related signaling pathway and extracellular matrix proteins enhanced the viability, proliferation, and migration of NSPC, and thus promoted the differentiation of NSPC into neurons.
Sun et al.	2019	LNCs, NNCs	Nothing	After implantation, inflammation and scarring were reduced, ENSCs were activated and migrated to damaged tissue, and nerve fiber growth was increased.

Abbreviations: BMSC, bone marrow mesenchymal stem cell; CPH, conductive polymer hydrogel; ENSC, endogenous neural stem cell; LOCS, linear ordered collagen scaffold; LNC, ladder‐like porous channel wall; NNC, nanofibrous channel wall; NSPC, neural stem/progenitor cells; PTX, Paclitaxel; SCI, spinal cord injury.

## 6 CONCLUSIONS AND PROSPECTS

**Figure 3:**
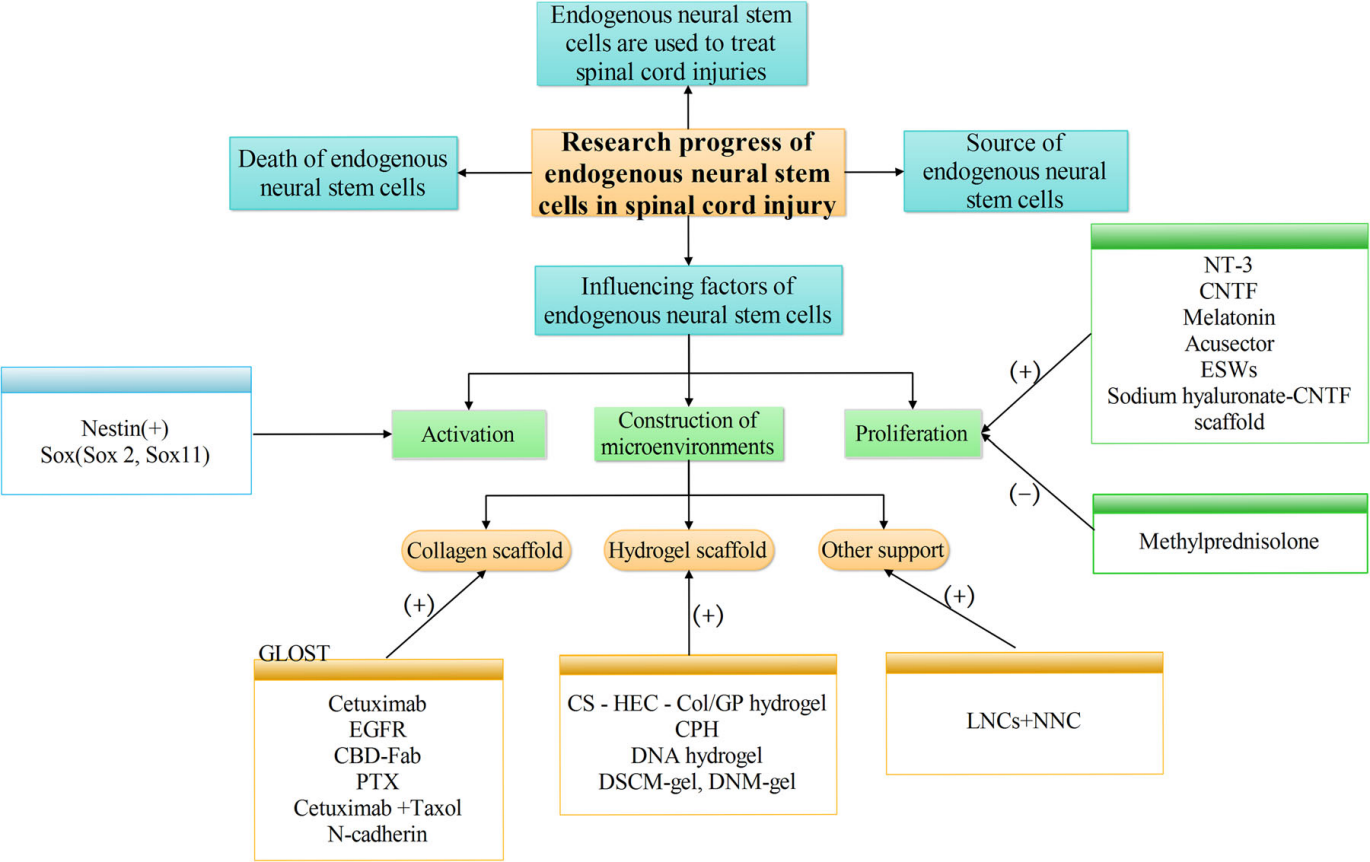
Mind map of the full text. CNTF, ciliary neurotrophic factor; CPH, conductive polymer hydrogel; EGFR, epidermal growth factor receptor; ESW, extracorporeal shock wave; LNC, ladder‐like porous channel wall; NNC, nanofibrous channel wall; and PTX, Paclitaxel. [Color figure can be viewed at wileyonlinelibrary.com]

Since the first discovery of stem cells in the 1960s, neural stem cell therapy, as a new treatment method for neurological diseases, has attracted considerable attention. ENSCs are of vital importance for the treatment of SCI because they improve nerve function and promote nerve regeneration. However, previous studies have shown that the central spinal canal remains open only in early childhood and gradually becomes occluded with age, replaced by astrocyte proliferation, ependymal cell clusters, and perivascular pseudoknots. The ependymal residue did not proliferate and differentiate even under the stimulation of SCI. From zebrafish to mice and rats, ENSCs seem to play an important role on SCI repair; however, they are not present in humans. This may be why many therapeutic approaches succeed in experiments, but fail in the clinic. It is worth noting that the current hot research topic focuses on how to activate ENSCs and guide them to migrate to the injury site to proliferate and differentiate into neurons with repair functions. Current studies have shown that many factors affect the activation and proliferation of ENSCs, including various neurotrophic factors, hormones, and physical therapy methods. Nestin and *Sox* not only serve as markers of ENSCs but also promote their activation. Hormones such as melatonin and methylprednisolone can significantly promote the proliferation of ENSCs. Physical therapy such as electroacupuncture and ESWs also play an important role in promoting the proliferation of ENSCs. Furthermore, determination of the optimal treatment plan from the numerous influencing factors is a major problem. Also, the construction of the microenvironment after SCI is one of the important factors affecting the proliferation and differentiation of ENSCs. The destruction of the microenvironment after SCI led most spinal ENSCs to develop into astrocytes and form glial scars, while only a small proportion of ENSCs differentiated into neurons. Future research direction will focus on ways to improve the microenvironment of ENSCs, increase neurogenesis, and protect functional newborn neurons and mature neurons to prevent delayed apoptosis. Stent implantation has become a good solution, and various types of stents have been developed. However, the most effective scheme has not been decided yet (Figure 3), and further discussion and research are needed to contribute to the clinical treatment of SCI.

## AUTHOR CONTRIBUTIONS

Ya‐Ting Wang wrote the article. Hao Yuan conceptualized the topic and revised the article.

## CONFLICT OF INTEREST

The authors declare no conflict of interest.

## TRANSPARENCY STATEMENT

Ya‐Ting Wang and Hao Yuan affirm that this manuscript is an honest, accurate, and transparent account of the study being reported, that no important aspects of the study have been omitted, and that any discrepancies from the study as planned (and, if relevant, registered) have been explained.

## ETHICS STATEMENT

Not applicable.

## Data Availability

Data sharing is not applicable to this article as no new data were created or analyzed in this study.
